# Challenges and opportunities in alcohol screening and brief interventions in new settings: A Narrative Review of Implementation Initiatives

**DOI:** 10.1186/1940-0640-10-S2-O39

**Published:** 2015-09-24

**Authors:** Niamh Fitzgerald

**Affiliations:** 1 Institute for Social Marketing, UK Centre for Tobacco and Alcohol Studies, School of Health Sciences, University of Stirling, Stirling, UK

## Background

Alcohol screening and brief interventions (SBI) have a history and good evidence of efficacy in primary care settings [1]. Efficacy evidence is variable across other settings and much is unknown including mechanisms of action, and optimal screening or implementation approaches[2,3]. Despite this, implementation outside of primary care has had much attention including in non-health settings, particularly in the UK [4-6].

This study aimed to discuss, and present for debate, challenges and opportunities relating to alcohol SBI in new settings from published and previously unpublished studies of recent SBI implementation in Scotland and England.

## Material and methods

A narrative review was conducted of evidence from diverse studies including research into training and implementation of alcohol SBI outside of primary care (accident and emergency; antenatal; social care; community/mental health; homelessness and multidisciplinary teams).

## Results

The challenges and opportunities can be conceptualised in terms of questions that should be asked before considering implementation of alcohol brief interventions in a new setting: (1) Is there a need for SBI delivery in this service? (2) How are practitioners currently addressing alcohol use - how when, where does SBI fit in? (3) Will SBI work/do harm in this setting? (4) Will SBI be perceived as legitimate by practitioners and acceptable to service-users? (5) Will practitioners have the ability to deliver SBI? (6) What support will be needed? (7) What will support routine implementation of SBI in this setting?

## Conclusion

Careful consideration should be given to every aspect of the design, purpose, context and evaluation of alcohol SBI in any given setting preferably by robust stepwise research prior to widespread implementation. Avoiding assumptions, including about screening methods and intervention goals, is likely to be important for effectiveness, implementation and avoiding unintended harms.
